# Social Image Security with Encryption and Watermarking in Hybrid Domains

**DOI:** 10.3390/e27030276

**Published:** 2025-03-06

**Authors:** Conghuan Ye, Shenglong Tan, Jun Wang, Li Shi, Qiankun Zuo, Wei Feng

**Affiliations:** 1 School of Information Engineering, Hubei University of Economics, Wuhan 430205, China; p2pgrid@hbue.edu.cn (C.Y.); tsl@hbue.edu.cn (S.T.); wjstky@hbue.edu.cn (J.W.); sl@hbue.edu.cn (L.S.); qk.zuo@hbue.edu.cn (Q.Z.); 2 Hubei Key Laboratory of Digital Finance Innovation, Hubei University of Economics, Wuhan 430205, China; 3 Hubei Internet Finance Information Engineering Technology Research Center, Hubei University of Economics, Wuhan 430205, China; 4 School of Mathematics and Computer Science, Panzhihua University, Panzhihua 617000, China

**Keywords:** multimedia social platform, social image security, joint watermarking and encryption, hopfield neural network, Game of Life

## Abstract

In this digital era, social images are the most vital information carrier on multimedia social platforms. More and more users are interested in sharing social images with mobile terminals on multimedia social platforms. Social image sharing also faces potential risks from malicious use, such as illegal sharing, piracy, and misappropriation. This paper mainly concentrates on secure social image sharing. To address how to share social images in a safe way, a social image security scheme is proposed. The technology addresses the social image security problem and the active tracing problem. First, discrete wavelet transform (DWT) is performed directly from the JPEG image. Then, the high-bit planes of the LL, LH, and HL are permuted with cellular automation (CA), bit-XOR, and singular value decomposition (SVD) computing, and their low-bit planes are chosen to embed a watermark. In the end, the encrypted and watermarked image is again permuted with cellular automation in the discrete cosine transform (DCT) domain. Experimental results and security analysis show that the social image security method not only has good performance in robustness, security, and time complexity but can also actively trace the illegal distribution of social images. The proposed social image security method can provide double-level security for multimedia social platforms.

## 1. Introduction

The rapid development of mobile social networks has made social image sharing popular. Social image sharing refers to the practice of individuals with social connections sharing diverse types of images via public multimedia platforms. For instance, this includes personally generated images distributed on social media platforms, the sharing of medical images between doctors and their patients who have established social relationships, and the dissemination of surveillance images on security system network platforms. JPEG images, as the main form of social images, are distributed on multimedia social platforms ubiquitously [1]. Social image sharing underscores potential risks of privacy disclosure. Content sharing on multimedia social platforms causes distinctive security challenges due to the privacy concerns of users [2]. Privacy protection has become increasingly more important for social image sharing on multimedia social platforms [3].

Secure social JPEG image sharing is still in its infancy. Confidentiality and traceability are dependent on secure content sharing [4]. Thus, multi-level secure techniques [5,6] should be adopted. To obtain confidentiality, content should be changed into an unintelligible visual content. The encrypted form of multimedia content can deter illegal access during the content-sharing processes. To realize multimedia content encryption, chaotic systems can be used to encrypt images. Chaotic image encryption algorithms are an important topic in secure image distribution on multimedia social platforms [7,8]. A chaotic signal, which usually looks like noise, is the main advantage of chaotic systems [3,9,10,11].

Chaotic image encryption schemes have been previously proposed [12,13,14]. In addition, an image encryption algorithm [15] was presented based on a 2D chaotic map. Kocak et al. proposed a color image encryption method [16]. Feng et al. proposed an image encryption algorithm based on fusion strategy [17], a multi-image encryption method [18], and an image encryption algorithm using a hyper-chaotic map [19]. A color image encryption scheme was proposed in [20]. Face image security algorithms were researched in [21,22], and an encryption method for ship images was proposed in [23]. An image encryption scheme consists of shuffle and block XOR [24]. The encryption algorithm in [25] includes cyclical bit shift and XOR operations. However, these security schemes only focus on content encryption. Once the encrypted social multimedia content is received and decrypted, the protection no longer exists. The decrypted plaintext could be copied and redistributed to a third party. Therefore, the multimedia content will not be protected, and the privacy may still be leaked.

To deter content redistribution, digital watermarking/fingerprinting can be adopted [26,27] even though both encryption and watermarking can individually protect social images. To indefinitely protect multimedia content, both techniques are used for secure content sharing [28]. Their combination is therefore increasing [29,30,31,32]. However, due to the large volume of multimedia data, the efficiency of content sharing is low if the encryption procedure is conducted in the spatial domain.

Existing image encryption algorithms are not suitable for social JPEG image sharing because of the resource-constrained mobile terminal used. Millions of users can share images/videos on multimedia social platforms taken with professional cameras and personal smartphones. To avoid computing and storage resource consumption, images are compressed before they are uploaded to multimedia social platforms [33]. Although watermarking can authenticate copyright, watermarking in the encrypted compressed domain is still in its infancy. Joint watermarking and encryption (JWE) can provide a more comprehensive security level for social multimedia. The combination of a watermark and encryption has facilitated studies regarding privacy protection and multimedia security. For existing JWE schemes, issues regarding the multimedia big data effect still exists.

This research mainly focuses on the privacy leakage of social multimedia. The authors address the issue of social image secure sharing. The authors also research new challenges of social JPEG image distribution. A novel social multimedia dissemination algorithm is proposed based on a hierarchical community structure to deal with privacy concern issues. The paper proposes a social image security algorithm with Game of Life (GoL) confusion, bit-XOR, and singular value decomposition (SVD) diffusion for wavelet coefficients, which are directly obtained from discrete cosine transform (DCT) blocks.

In this paper, discrete wavelet transform (DWT) from DCT blocks can help to lower the algorithm complexity. Scrambling based on GoL can balance social image security and efficiency. The proposed social image security scheme can solve the problems that existing methods face. The main highlights are as follows:

(1) DWT from block DCTs can encrypt and watermark images in hybrid domains.
(2) The algorithm that protects social JPEG images is based on DWT from DCT blocks.
(3) A lightweight security approach for social multimedia platforms is beneficial for resource-constrained equipment.

There is no related research for privacy protection with GoL permutation in hybrid domains. The background for this paper is presented in Section 2. Section 3 presents details of the security algorithm. Then, Section 4 shows the experimental results and analyzes the security. Conclusions and future work are both presented in Section 5.

## 2. Background

### 2.1. Singular Value Decomposition

The SVD [34] is a factorization and approximation technique that is highly useful in linear algebra. It is capable of performing an optimal matrix decomposition in a least-squares sense for any given matrix. Typically, an image can be regarded as a matrix *A* with size M×N, which has non-negative scalar entries. Subsequently, *A* can be decomposed using SVD. As a useful tool in signal processing, SVD of *A* can be represented as follows:

(1) $$A=USV^{T}$$

where *A* is a rectangular matrix. Both *U* and *V* are orthogonal and unitary matrices. *S* is a singular value matrix. *T* represents the conjugate transpose operation. Both *U* and *V*, when multiplied by their respective transpose matrices, yield the identity matrix.

### 2.2. The Tree Structure Haar Transform

The TSH wavelet transform, as a hierarchical structure transform, has a good correspondence with social fingerprinting. Digital watermarking techniques using discrete wavelet transform have much higher robustness. The discrete wavelet transform divides an image into four components: LL, HL, LH, and HH sub-bands. The last three of these components are detail sub-components, while the approximate component is very similar to the original image. The discrete wavelet transform is formed by low-pass filtering in both row and column directions, and the components can be further decomposed. In order to make the algorithm scalable, the non-binary tree structure Haar (TSH) [35] transform is chosen, and this TSH wavelet transform can be adapted to the user’s multi-level social fingerprint code. According to the hierarchical structure of the fingerprint code, the corresponding discrete point vector (DPV) is determined to assist the tree structure Haar wavelet transform, and a mapping relationship is established between the carrier and the fingerprint code, which enables the code segments of the multi-level fingerprint code to be embedded into the low-bit planes of coefficients in the TSH domain in parallel and avoids the duplication of embedding the community code segments. This embedding mode can effectively alleviate the server-side duplication of embedding the entire social fingerprint code. The overhead of repeated embedding of the whole fingerprint codeword at the server side can be effectively reduced.

**Figure 1:**
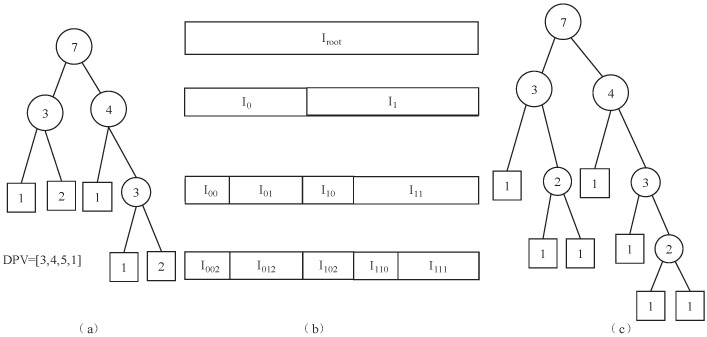
The DPV production scheme: (**a**) labeled binary tree; (**b**) the interval corresponding to the tree in (**a**); (**c**) the corresponding complete binary tree.

Given an interval *I*, which is split into $I_{0}^{H}=\left[1,2^{-1}N\right]$ and $I_{1}^{H}=\left[2^{-1}N+1,N\right]$ according to Figure 1, the TSH function is defined as follows:

(2) $$I_{0}^{H}=\left[1,2^{-1}N\right]$$

(3) $$\begin{array}{c} \Psi^{H}\left(t\right)=\left\{\begin{array}{c} \frac{1}{\sqrt{N}}\;\;\;\;\;\;\;\;\;\;\;\;\;\;\;\;\;\;\;\;\;\;\;\;\mathrm{for}\;\;\;t\in I_{0}^{H}\\ -\frac{1}{\sqrt{N}}\;\;\;\;\;\;\;\;\;\;\;\;\;\;\;\mathrm{for}\;\;\;t\in I_{1}^{H}\\ 0\;\;\;\;\;\;\;\;\;\;\;\;\;\;\;\;\;\;\;\;\;\;\;\;\;\;\;\;\;\;\;\;\;\;\mathrm{otherwise} \end{array}\right. \end{array}$$

where both $\left|\right|\Psi^{H}\left(t\right)\left|\right|=1$ and $\Psi^{H}\left(t\right)$ are orthogonal, and ||•|| denotes a norm. Usually, the interval *I* can be partitioned into $I_{0}^{TSH}=\left[1,v_{0}\right]$ and $I_{1}^{TSH}=\left[v_{0}+1,N\right]$, where $1\leq v_{0}<N$. The function $\Psi^{TSH}\left(t\right)$ can be defined as follows:

(4) $$\begin{array}{c} \Psi^{TSH}\left(t\right)=\left\{\begin{array}{c} \sqrt{\frac{v_{1}}{v_{0}N}}\;\;\;\;\;\;\;\;\;\;\;\;\;\;\;\;\;\;\;\;\;\;\;\;\mathrm{for}\;\;\;t\in I_{0}^{TSH}\\ -\sqrt{\frac{v_{0}}{v_{1}N}}\;\;\;\;\;\;\;\;\;\;\;\;\;\;\;\mathrm{for}\;\;\;t\in I_{1}^{TSH}\\ 0\;\;\;\;\;\;\;\;\;\;\;\;\;\;\;\;\;\;\;\;\;\;\;\;\;\;\;\;\;\;\;\;\;\;\mathrm{otherwise} \end{array}\right. \end{array}$$

where $v_{1}=N-v_{0}$. This construction can be iterated by splitting $I_{0}^{TSH}$ into $I_{00}^{TSH}$ and $I_{01}^{TSH}$, and $I_{1}^{TSH}$ into $I_{10}^{TSH}$ and $I_{11}^{TSH}$. All functions $\Psi^{TSH}\left(t\right)\left(1,N,v_{0}\right)$ are orthogonal TSH functions.

Assume a social image *I* has size (S×K)×(S×L). Then, *I* is partitioned into K×L patches. $BL_{lk}$ denotes the lk_th block. The size of each patch is S×S, and its corresponding DCT coefficient patch is denoted as $C_{lk}\left(u,v\right)$.

In this paper, a social JPEG image is partially decoded to obtain DCT blocks. All of the DCT blocks are used to compute one-level DWT coefficients. The most significant aspect of the wavelet transform is that a mapping can be established between the tree structure of wavelet decomposition and the hierarchical fingerprint code based on social network analysis (SNA).

The 2D DCT transform can be performed on $BL_{lk}$, resulting in $C_{lk}\left(u,v\right)=B_{1}\times BL_{lk}\times B_{1}^{T}$. Conversely, $BL_{lk}$ can be recovered by $BL_{lk}=B_{1}^{-1}\times C_{lk}\times {\left(B_{1}^{T}\right)}^{-1}$. Both $B_{1}$ and $B_{1}^{T}$ are block DCT matrices, and they are orthogonal.

Then, the DWT derived from DCT blocks is as follows:

(5) $$KR=H\times I\times Q^{T}$$

where *H* and *Q* are coefficient transformation matrices, both of which are orthogonal and invertible. *H* contains $h_{z}\left(k\right)$, and it is a Haar basis function.

$I=H^{T}\times KR\times Q$ is the inverse Haar wavelet transform. With the inverse transform, coefficient matrix KR in the DWT domain can be expressed as

(6) $$KR=A_{1}\times C_{part}\times A_{2}$$

where $A_{1}=H\times B_{1}^{-1}$, $A_{2}={\left(B_{1}^{T}\right)}^{-1}\times Q^{T}$, and KR represents the coefficient in the DWT domain. Through inverse transformation, the DCT blocks can be obtained as follows:

(7) $$C_{part}=A_{1}^{T}\times KR\times A_{2}^{T}$$

### 2.3. Cellular Automation

CA [36] is a complex spatial–temporal system that is a time discrete system. The two-dimensional CA is known as the Game of Life (GoL). The seed matrix of GoL has a size M×N, and each cell in the grid can be in one of two states: 0 represents dead; 1 represents alive. Chaotic behavior can be generated through CA. The GoL has simple operations for changing the alive and dead states of cells, resulting in low computational complexity. This low-complex operation makes it an effective method for encrypting social images. In the two-dimensional GoL matrix, each cell has eight neighbors in the seed matrix, located either horizontally, vertically, or diagonally. Each cell evolves according to the following GoL rules:

Rule 1: Cells that are alive but have fewer than two live neighbors will die.
Rule 2: Cells that are alive and have two or three live neighbors will survive.
Rule 3: Cells that are alive but have more than three live neighbors will die.
Rule 4: Cells that are dead but have exactly three live neighbors will revive.

**Figure 2:**
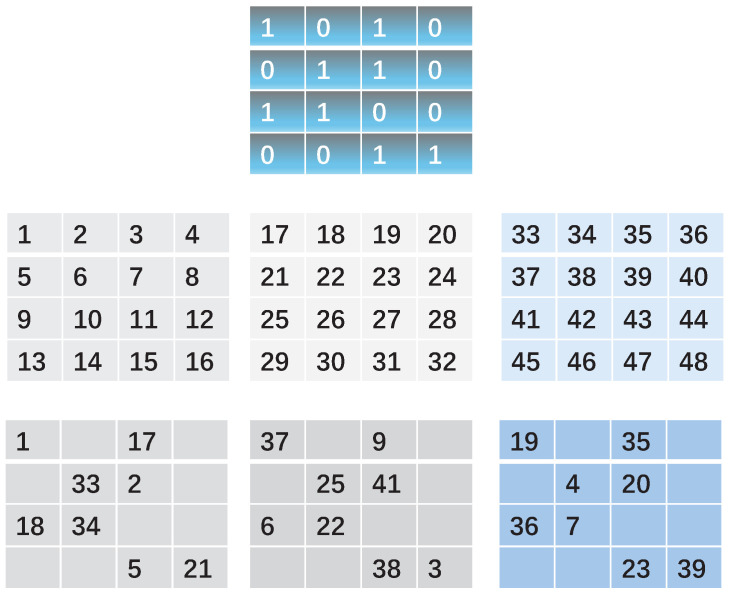
The GoL scheme: superior, the initial seed of GoL; middle, the original images; inferior, the scrambled images.

The evolution of GoL is based on an orthogonal grid of cells. The process is illustrated in Figure 2. The main steps are as follows:

(1) A random sequence ($x_{1}x_{2}\cdots x_{M\times N}$) is produced using a given chaotic map. A grid of cell $G^{0}$ is created based on this random sequence. The sum *S* of the sequence is calculated as $S=sum(x_{1}+\ldots +x_{M\times N})$, and the mean value MV is calculated as MV=S/(M×N). For each cell in the grid, if the corresponding value $x_{i}>MV$, then the cell is set to 1, otherwise, it is set to 0. Using the initial grid $G^{0}$ and the rules of GoL, a sequence of matrices $\left\{G_{1},G_{2},\ldots ,G_{k}\right\}$ is generated.
(2) According to the index, the elements of the original images are arranged in a zigzag formation and sequentially insert them into the encrypted images.

Figure 2 shows the GoL process for three images. The GoL permutation operation is based on zigzag order applied to the elements within these three images.

### 2.4. Secure Hash Algorithm (SHA-3)

SHA-3 can process messages of any length up to a predefined limit [37]. It provides data integrity and security while also generating random sequences. As a popular hash function, the SHA-3 algorithm can generate 256-bit or 512-bit hash values for any form of communication. In this study, the proposed SHA-3 method will utilize a 256-bit hash value. Even if the original message undergoes a very small change, the hash value of the changed message will be completely different. Furthermore, since the hash computing relies on bit-level operations, it provides superior temporal performance. Due to it being very sensitive to the initial input content, it is widely used for key generation.

### 2.5. Chaotic System

Recently, many 2D chaotic maps have been designed for image encryption [15,16,38]. However, despite their complex chaotic trajectories, these chaotic maps often suffer from low efficiency due to their complex structures. In this paper, we adopted a chaotic map [17] for image encryption as follows:

(8) $$\left\{\begin{array}{c} u_{i+1}=\left(\alpha u_{i}^{2}+10^{\beta }v_{i}\right)\;\mathrm{mod}\;1,\\ v_{i+1}=\left(\alpha v_{i}^{2}+10^{\beta }u_{i}\right)\;\mathrm{mod}\;1, \end{array}\right.$$

where $u_{0}$ and $v_{0}$ are the initial values for the chaotic map, while $u_{i}$ and $v_{i}$ are the corresponding outputs (i=1,2,…). Both α and β are control parameters.

The Hopfield neural network system [39] can demonstrate chaotic trajectories. The following is the 3D chaotic neural network system:

(9) $$\left\{\begin{array}{c} x_{1}^{\prime }=-x_{1}+2f\left(x_{1}\right)-f\left(x_{2}\right)\\ x_{2}^{\prime }=-x_{2}+1.7f\left(x_{1}\right)+1.7f\left(x_{2}\right)+1.1f\left(x_{3}\right)\\ x_{3}^{\prime }=-x_{3}-2.5f\left(x_{1}\right)-2.9f\left(x_{2}\right)+0.56f\left(x_{3}\right) \end{array}\right.$$

(10) $$f\left(x_{i}\right)=\tanh\left(x_{i}\right)\;i=1,2,3$$

where $f(x_{i})$ is the tangent function.

### 2.6. Joint Watermarking and Encryption

JWE can be utilized to protect privacy. A digital watermarking scheme involves embedding watermark information into a carrier. Recently, the need to apply watermarking in conjunction with image encryption for multimedia content protection has been increasing. There are two distinct methods for combining watermarking and encryption to protect multimedia content. The first method integrates watermarking with the image decryption operation on the client side. The second method primarily combines watermarking and encryption operations on the server side. Image encryption can either be full encryption, whereby all multimedia content is encrypted, or selective encryption, where only the crucial parts are encrypted.

## 3. The Proposed Scheme

The social image security scheme encompasses encryption and watermarking. Joint encryption and watermarking can offer dual-level protection. Furthermore, the hybrid transform domain allows for the embedding of fingerprints and encryption to be performed in parallel. Most social images are distributed in JPEG format in plaintext mode. JPEG images are highly popular on multimedia social platforms [40]. JPEG compression removes redundant data, achieving a high compression ratio [41]. To encrypt and watermark JPEG images in hybrid domains, partial decoding can be applied to JPEG images to obtain DCT blocks, on which DWT is then performed.

### 3.1. Coding Using Social Network Analysis

**Figure 3:**
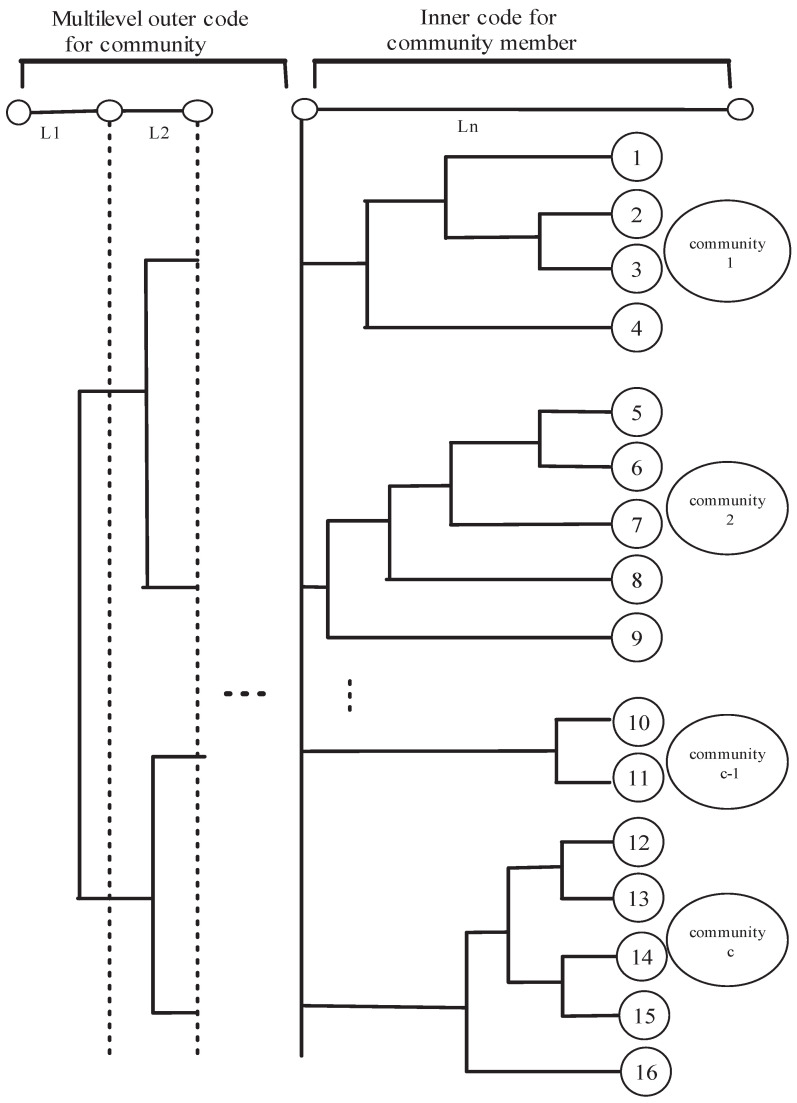
Encoding using social network analysis.

The fingerprint code is generated using SNA. According to Figure 3, the social network exhibits characteristics of a hierarchical community structure. Each individual user belongs to a community, and users within the same community share a common social relationship and a common community structure. Subsequently, the community to which they belong can be coded with the Tardos scheme, and each user within a community is assigned a user code based on the Tardos scheme [42]. Consequently, the fingerprint code possesses a hierarchical structure that mirrors the community structure.

### 3.2. The Joint Watermarking and Encryption Scheme

**Figure 4:**
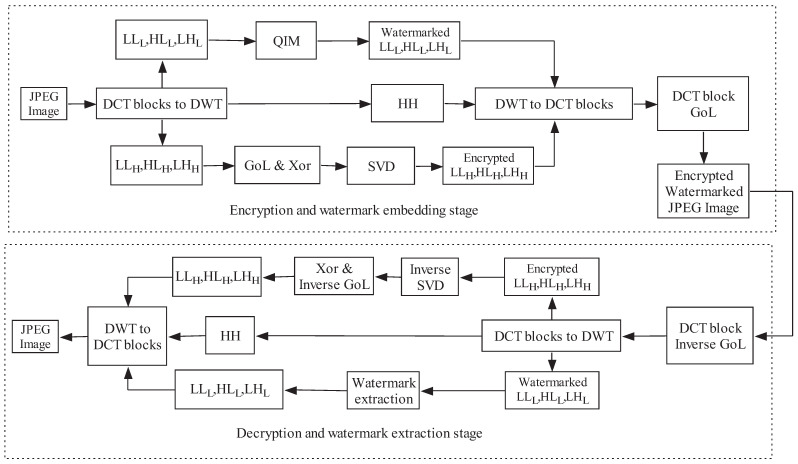
The proposed security scheme.

The JWE scheme incorporates selective encryption for the high-bit planes of the LL, HL, and LH sub-bands, along with watermarking for the low-bit planes of the LL, HL, and LH sub-bands. The JPEG image is partly decompressed into block DCTs, as illustrated in Figure 4. Subsequently, DWT is performed on these DCT blocks using a 1-to-1 map between DCT blocks and DWT. The fast DWT domain yields the LL, LH, HL, and HH sub-bands. The LL, HL, and LH sub-bands are then decomposed into high-bit planes and low-bit planes through coefficient separation. The high-bit planes undergo confusion based on GoL, followed by diffusion using bit-XOR operations and SVD computing. Meanwhile, the low-bit planes are watermarked using quantization index modulation (QIM). The application of DWT directly on block DCTs is efficient for encrypting and watermarking in hybrid domains, as it can avoid the time-consuming conversion between the spatial domain and the DCT/DWT domain.

#### 3.2.1. Fingerprint Embedding and Traitor Tracing

Figure 4 illustrates the watermarking scheme. The entire JWE process involves embedding fingerprints in the low-bit planes and encrypting data in the high-bit planes. In the first stage of JWE, which focuses on watermarking techniques for embedding fingerprints into digital images, it is advantageous to embed fingerprint information in the transform domain to enhance both robustness and perceptual quality. Due to its increasing popularity, the DWT is frequently utilized in recent watermarking schemes, where the DWT coefficients are modified.

For compressed JPEG images, we directly apply DWT to the block DCTs. Then, we embed hierarchical fingerprints into the hierarchical sub-band of the DWT domain using an improved quantization index modulation (QIM) scheme [43] in parallel. To preserve the visual quality of the marked social image, we adaptively quantize the low-bit planes of the coefficients in the LL, HL, and LH sub-bands using a customized quantization size.

Suppose there are $N_{u}$ users. The fingerprint embedding is based on QIM. Let $X^{O}=\left(x_{1},x_{2},\ldots ,x_{L}^{O}\right)$ represent the community segment of the codeword, which is embedded into the low-bit planes of the HL and LH sub-bands. The embedding scheme for the community segment is based on Equation (11). Additionally, we select the low-bit planes of the LL sub-band to obtain another vector, $X^{I}=\left(x_{1},x_{2},\ldots ,x_{L}^{I}\right)$, which is used to embed the inner segment of the fingerprint codeword. Both $X^{I}$ and $X^{O}$ have lengths corresponding to the inner and outer segments of the codeword, respectively. $L=L^{O}+L^{I}$ is the length of fingerprint codeword. The codeword embedding scheme is described in Equation (11).

(11) $$Y_{k}=Q_{\Delta }\left(X_{k}^{O}+W_{k}+d_{k}\right)-W_{k}-d_{k},k=1,2,\ldots ,N_{u}$$

where $Q_{\Delta }\left(•\right)$ represents a quantization function with a constant step size Δ. $X_{k}^{O}$ denotes the low-bit planes of the LH and HL sub-band, $W_{k}$ is a codeword for user *k*, and $d_{k}$ is a dither vector with a uniform distribution in the range (−Δ/2, Δ/2).

To detect illegal redistribution, the fingerprinted low-bit planes of the LL, HL and LH sub-bands are extracted. The user *m* identified as the traitor is determined according to Equation (12).

(12) $$\hat{m}=\arg\min_{k=1,2,\ldots N_{u}}{∥Z-Y_{k}∥}^{2}$$

#### 3.2.2. Encryption and Decryption Algorithm

Image encryption disrupts the original pixel values to hide information. Visual security is crucial for social image encryption, as it ensures that the cipher image is not perceived to contain any original information. In this case, the visual perception of the cipher image is secure. Without the correct key, the encrypted image cannot be decrypted, making it an effective method for protecting sensitive content. Resource-constrained mobile terminals are the primary medium for content sharing on multimedia social platforms. Therefore, it is essential to process social images in the compressed domain to minimize computational overhead. Processing social images in the spatial domain can result in longer total encryption and fingerprinting times, ultimately leading to big data problems. To address this, selective encryption in hybrid domains can ensure low computational complexity while avoiding redundant embedding of fingerprint information.

Selective encryption in hybrid domains is an effective approach for ensuring social image security. A JWE algorithm is proposed for secure sharing of social images in hybrid domains. The proposed JWE scheme is mainly based on GoL, bit-XOR, and SVD. High-bit planes and low-bit planes of wavelet coefficients are encrypted and watermarked in parallel to enhance security. The proposed coefficient encryption algorithm works as follows:

Step 1: Given a JPEG image *I*, perform DWT on DCT blocks of the image. Assume that the matrix *M* represents the HH sub-band of *I*. Partition *M* into two parts: $M_{1}$, $M_{2}$. Calculate the sum of the elements in both parts, denoted as $Sum_{M_{1}}$ and $Sum_{M_{2}}$, respectively. Obtain the threshold Th by subtracting $Sum_{M_{2}}$ from $Sum_{M_{1}}$: $Th=Sum_{M_{1}}-Sum_{M_{2}}$. Compute the SHA-3 hash of Th to obtain $V^{Th}=hash_{SHA-3}\left(Th\right)$. Partition $V^{Th}$ into 16 parts: $V_{1}^{Th}$, $V_{2}^{Th}$, …, $V_{16}^{Th}$. Initial values $x_{1}$, $x_{2}$, $x_{3}$, $u_{1}$, $v_{1}$, and control parameters α and β can be generated based on these hash values: $V_{1}^{Th}$, $V_{2}^{Th}$, …, $V_{16}^{Th}$.

(13) $$x_{1}=\frac{V_{1}}{2^{17}},x_{2}=\frac{V_{2}}{2^{17}},x_{3}=\frac{V_{3}}{2^{17}}$$

(14) $$u_{1}=\frac{V_{5}}{2^{17}},v_{1}=\frac{V_{6}}{2^{17}},\alpha =\frac{V_{7}}{2^{17}},\beta =\frac{V_{8}}{2^{17}}$$

Step 2: Regard the high-bit planes of wavelet coefficients in the LL, HL, and LH sub-bands as three different images. Scramble them based on GoL in Figure 2. Then, three scrambled matrices $SM_{LL}$, $SM_{HL}$, and $SM_{LH}$ can be obtained.

Step 3: Using the Hopfield neural network system, generate three chaotic sequences, denoted as $RP_{M\times N}^{J^{K}}=\left\{rp_{1}^{J^{K}},rp_{2}^{J^{K}},\ldots ,rp_{M\times N}^{J^{K}}\right\}$. Subsequently, obtain the ceiling values of these sequences to form $CRP_{M\times N}^{J^{K}}=\left\{crp_{1}^{J^{K}},crp_{2}^{J^{K}},\ldots ,crp_{M\times N}^{J^{K}}\right\}$, where $crp_{i}$=ceiling($rp_{i}$). Based on these ceiling sequences $CRP_{M\times N}^{J^{K}}$, we can then construct three random matrices: $RM_{LL}$, $RM_{HL}$, and $RM_{LH}$.

(15) $$C_{k}=⊕(SM_{k},RM_{k})$$

where k={LL,LH,HL}.

Step 4: Using the Hopfield neural network system, generate three chaotic sequences, denoted as $FP_{M\times N}^{J^{K}}=\left\{fp_{1}^{J^{K}},fp_{2}^{J^{K}},\ldots ,fp_{M\times N}^{J^{K}}\right\}$. Subsequently, obtain the ceiling values of these sequences to form $CP_{M\times N}^{J^{K}}=\left\{cp_{1}^{J^{K}},cp_{2}^{J^{K}},\ldots ,cp_{M\times N}^{J^{K}}\right\}$, where $cp_{i}$=ceiling($fp_{i}$). Arrange $CP_{M\times N}^{J^{K}}=\left\{cp_{1}^{J^{K}},cp_{2}^{J^{K}},\ldots ,cp_{M\times N}^{J^{K}}\right\}$ into an M×N matrix denoted as $CP_{k}$. Next, conduct SVD on $CP_{k}$, resulting in $CP_{k}=U_{CP_{k}}S_{CP_{k}}V_{CP_{k}}^{T}$, where *k* can be {LL,LH,HL} to indicate different matrices.

Step 5: Diffuse the confused and bit-XORed high-bit planes of the wavelet coefficients in the LL, HL, and LH sub-bands using the orthonormal matrices $U_{CP_{k}}$ and $V_{CP_{k}}^{T}$, as

(16) $$I_{K}^{E}=\left\{\begin{array}{c} U_{CP_{k}}C_{k}V_{CP_{k}}^{T},\;\;\;M\leq N\\ V_{CP_{k}}C_{k}U_{CP_{k}}^{T},\;\;\;M>N \end{array}\right.$$

where k={LL,LH,HL}.

Step 6: Combine the encrypted high-bit planes denoted as $I_{k}^{E}$, k={LL,LH,HL}, with the watermarked low-bit planes, denoted as $Y_{k}$, k={LL,LH,HL}, to obtain the encrypted and watermarked LL, LH, and HL sub-bands.

Step 7: Perform DWT to DCT block transform with the encrypted and watermarked coefficients. Then, apply GoL with a chaotic sequence ($y_{1}y_{2}\cdots y_{M/8\times N/8}$) generated by the 2D chaotic map to obtain confused DCT blocks. The result is the encrypted and watermarked image $I^{JWE}$.

The image decryption operation is the inverse process of encryption, and the decryption algorithm is as follows:

Step 1: Apply the inverse GoL to the watermarked and encrypted image $I^{JWE}$ to obtain the DCT blocks.

Step 2: Perform DWT decomposition to the DCT blocks to obtain LL, LH, HL, and HH sub-bands.

Step 3: Perform the inverse SVD operation on the diffused high-bit planes of the encrypted sub-bands, as follows:

(17) $${\tilde{I}}_{K}^{JWE}=\left\{\begin{array}{c} U_{CP_{k}}^{T}{\hat{I}}_{k}^{JWE}V_{CP_{k}},\;\;\;M\leq N\\ V_{CP_{k}}^{T}{\hat{I}}_{k}^{JWE}U_{CP_{k}},\;\;\;M>N \end{array}\right.$$

Step 4: Apply the bit-XOR operation to the inverted SVD content to obtain the confused coefficient matrix.

Step 5: Use the index matrix obtained during the GoL process to reverse the confusion in the coefficient matrix.

Step 6: Apply IDWT to the decrypted coefficients to retrieve the watermarked image $I^{W}$.

## 4. Performance Test and Comparative Analysis

**Figure 5:**
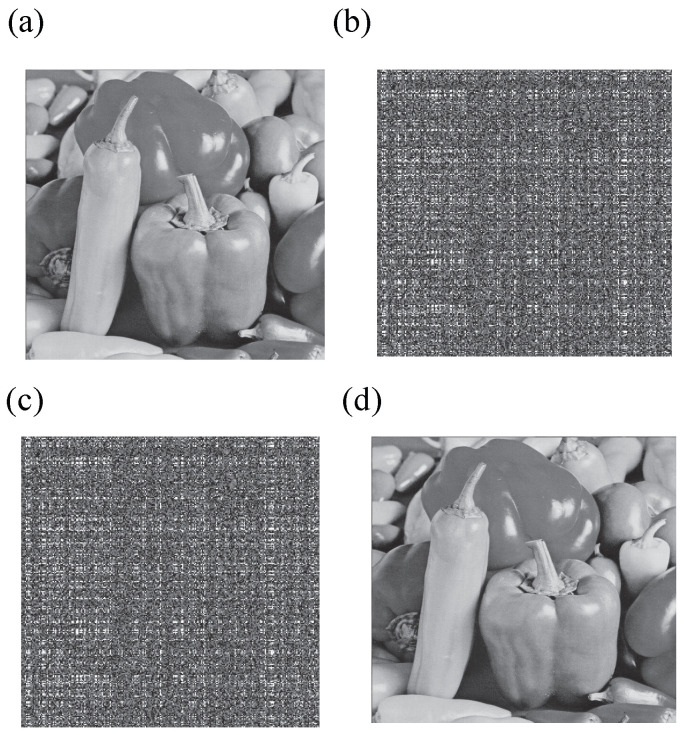
Encrypted and decrypted results: (**a**) The plain image. (**b**) The cipher image. (**c**) The reconstructed image with a different initial value. (**d**) The reconstructed image with correct parameters.

We implement the proposed social security scheme in MATLAB 2012. We used six types of test images (“Baboon, Peppers, Fishing boat, Airplane, Couple and Watch”, 512×512). Figure 5b shows the encrypted images. Figure 5d demonstrates the decrypted and watermarked image with fingerprint information. From Figure 5b, the visual security can be ensured.

### 4.1. Fingerprint Imperceptibility

The fingerprint is embedded into the social image during the encryption process. Specifically, the fingerprint information is embedded into the low-bit planes while the high-bit planes of the LL, LH, and HL sub-bands are being encrypted. To maintain high visual quality of the watermarked images, the fingerprint information should remain imperceptible or undetected in the fingerprinted copy. Related watermarked results are shown in Figure 5d. It is evident that the visual quality of the marked image has not undergone any noticeable change. Watermark in the decrypted images should not be perceived in Figure 5d. It is apparent that the security scheme possesses active tracing capabilities for future use.

### 4.2. Perceptual Effect of Encrypted Images

**Figure 6:**
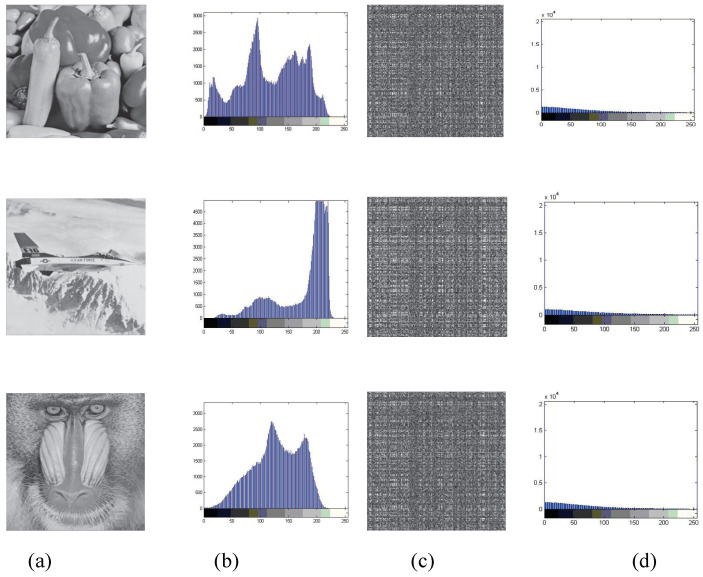
Experimental results: (**a**) Original images. (**b**) Initial histograms. (**c**) Encrypted images. (**d**) Encrypted histograms.

To protect social images from unauthorized access, encryption is necessary. An algorithm is considered to have a higher level of security if the encrypted images are unviewable to illegal users. Therefore, to limit illegal access, the visual quality of the encrypted images should be poor, as this indicates a safer encryption algorithm. Additionally, the watermark information should remain imperceptible in the decrypted and watermarked image. To achieve this, high-bit planes of the LL, HL, and LH sub-bands are selected for confusion with GoL, and inverse bit-XOR and SVD computing are utilized to diffuse the confused content for enhanced security. Figure 6c displays the experimental results, showcasing encrypted images that resemble noise-like signals and are thus unintelligible. This demonstrates the high security level of the encryption method. Furthermore, Figure 6c demonstrates the encrypted images obtained using the proposed selective encryption in hybrid domains. All encrypted images in Figure 6c are imperceptible, confirming the effectiveness of the proposed JWE algorithm in hybrid domains for social image security.

### 4.3. Exhaustive Attack Discussion

The keys for the selective encryption algorithm are generated using the SHA-3 hash method. The total keys include initial values $x_{1}$, $x_{2}$, $x_{3}$, $u_{1}$, $v_{1}$, and control parameters α and β. Assume the key sensitivity is $10^{-15}$; the 3D chaotic map is utilized twice in the encryption process. Consequently, the total key space is approximately $10^{15\times 10}=10^{150}$. This vast key space provides the selective encryption algorithm with a high level of resistance against brute-force attacks.

### 4.4. Statistical Attack Discussion

Statistical analysis attack is a method employed by cryptanalysts to decode a ciphertext by examining the statistical patterns within the encrypted text. Two common types of statistical attacks are histogram analysis and correlation analysis of adjacent pixels.

#### 4.4.1. Histogram Attack Discussion

Histogram attack analysis is a valuable tool for assessing the robustness of image encryption algorithms. In the proposed scheme, selective encryption in the wavelet domain is utilized specifically for JPEG images. To evaluate the effectiveness of the encryption algorithm, a comparison is made between the histograms of the encrypted images and the original images. If the encrypted histograms are uniformly distributed and significantly different from the corresponding original histograms, it indicates that the encryption algorithm is resilient to histogram attacks. Figure 6 provides visual evidence of this, where panels (b) and (d) display the gray-scale histograms of the original and encrypted images, respectively. From Figure 6, it is evident that the pixel values in the original images are concentrated in specific ranges. In contrast, the encrypted histograms in panel (d) exhibit a more uniform distribution and are markedly distinct from the original histograms. This suggests that the encryption algorithm has successfully obscured the statistical properties of the original images, making it difficult for an adversary to launch successful histogram attacks.

#### 4.4.2. Correlation Analysis

**Figure 7:**
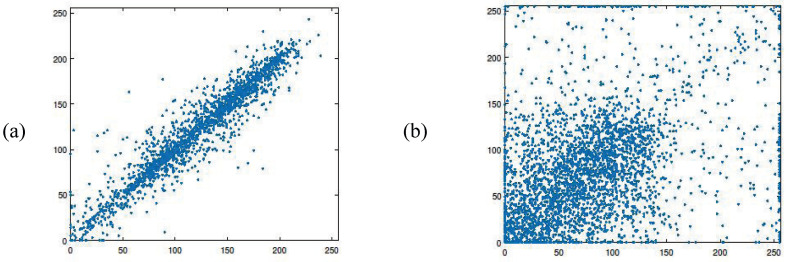
Correlation: (**a**) original image; (**b**) encrypted image.

The correlation analysis is used to evaluate the correlation between adjacent elements. Social images typically exhibit strong correlations among adjacent pixels. The proposed selective encryption algorithm in hybrid domains should be capable of disrupting these correlations. Figure 7a,b display the correlation of two adjacent pixels in the plain images and their corresponding encrypted images, respectively. Figure 7b demonstrates that the correlations in the encrypted image are greatly reduced. Essentially, the correlations present in the original image have been effectively destroyed by the encryption algorithm.

### 4.5. Encryption Process Discussion

**Figure 8:**
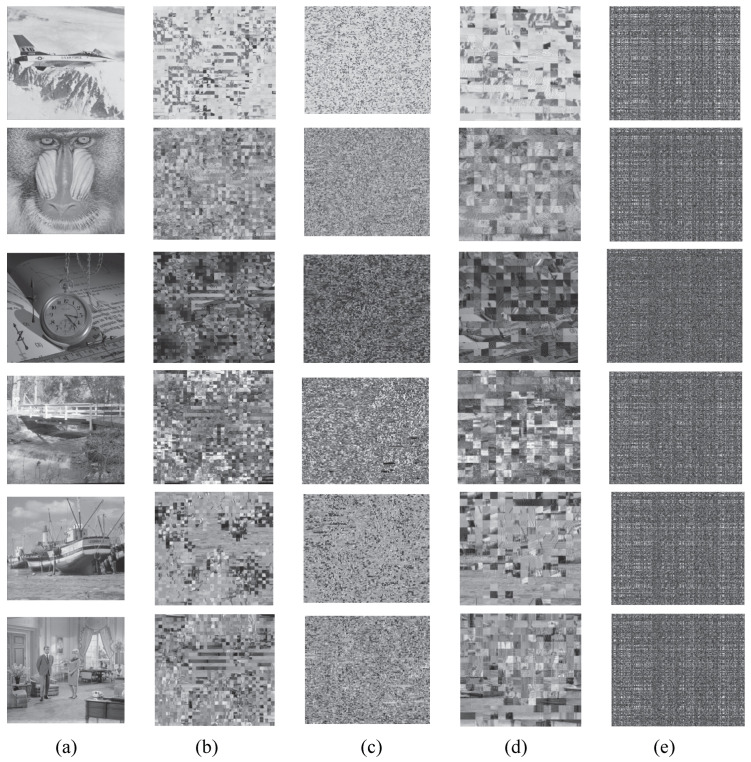
Encryption process discussion: (**a**) original images; (**b**) 4×4 block permutation across the high-bit planes of the LL, LH, and HL sub-bands; (**c**) single-coefficient permutation across the high-bit planes of the LL, LH, and HL sub-bands; (**d**) bit-XOR diffusion after 8×8 block permutation across the high-bit planes of the LL, LH, and HL sub-bands; and (**e**) SVD diffusion of (**d**).

The suggested hybrid watermarking and encryption approach encrypts critical coefficients. Figure 4 depicts the proposed algorithm. First, the high-bit planes of the LL, LH, and HL sub-bands are selected for scrambling. The scrambled results are demonstrated in Figure 8. Figure 8b,c show encrypted images with a 4×4 coefficient block scrambling based on GoL and single-coefficient GoL disruption across the LL, HL, and LH sub-bands. The unintelligibility can be enhanced by single-object permutation. Coefficient confusion based on GoL across the LL, HL, and LH sub-bands can achieve confusing and diffusion. Only confusion for the high-bit planes of the LL, LH, and HL sub-bands produces incomprehensible encryption effects. Figure 8 demonstrates that all permuted images are not perceptive. However, GoL permutation across the high-bit planes of the LL, LH, and HL sub-bands may not satisfy higher-level security requirements in some application areas. Therefore, in the proposed social image security scheme, bit-XOR and SVD diffusion operations are added after the GoL scrambling process, as shown in Figure 4. Figure 8d,e demonstrate encrypted images. Figure 8d shows the experimental results of bit-XOR diffusing after 8 coefficient block GoL permutation. Figure 8e shows the encrypted images after applying SVD diffusion to the encrypted images shown in Figure 8d.

All GoL permutations across the LL, HL, and LH sub-bands can render the permuted images unintelligible. In addition, the subsequent bit-XOR and SVD processing steps can provide sufficient security for the high-bit planes of the LL, LH, and HL sub-bands. If the GoL permutation across the high-bit planes of these sub-bands is cracked, the diffusion based on bit-XOR and SVD processing can still safeguard the encrypted social images. Therefore, the proposed technique is perceptually efficient. It can meet various levels of security requirements.

### 4.6. Encryption and Watermarking Performance Discussion

For the security of multimedia social platforms, it is essential to encrypt social images. It is crucial to achieve a balance between algorithm performance and security to meet the varying security level requirements on multimedia social platforms. The efficiency of the algorithm must be evaluated carefully. Given the resource constraints of many devices, if the social image security scheme takes too much time, it will not be a viable security method for multimedia social platforms.

#### 4.6.1. Comparative Analysis

In this section, we evaluate the efficiency of the encryption algorithm. For social image sharing, if the proposed selective encryption algorithm in hybrid domains has a high running time complexity, it will not be an effective solution for ensuring social image security. Conversely, if the proposed selective encryption algorithm has a low running time complexity and is capable of providing double-level security services within a strict time limit, the security scheme is considered efficient.

The JWE scheme is compared with some existing related algorithms [24,25]. Due to their comprehensive encryption approach, these algorithms are extremely time-consuming in the spatial domain, making full encryption impractical for social image sharing.

The image encryption method [24] consists of shuffling and block XOR operations. Rostami et al.’s image encryption process [24] involves three main steps. Firstly, the chaotic matrix is generated by one-dimensional chaotic mapping, and the image is divided into many blocks, and the values of the chaotic matrix are used to perform XOR operations with the values in each block. Secondly, one-dimensional logical mapping is employed to generate a matrix with the same dimensions as the image. This matrix is used to disturb the position of the image pixels. Finally, a new chaotic matrix is generated using a new initial value. This matrix is then used to perform XOR operations with the blocks of the image. Encryption algorithm in [25] incorporates cyclic bit shifts and XOR operations. Diaconu et al. proposed an encryption technology that combines cyclic displacement and chaos [25]. This technology is primarily divided into two steps. Firstly, each pixel value of the original image is converted into an 8-bit binary array. The number of ‘1’s in each binary array is then counted. The remainder is determined when this count is divided by 2. If the remainder is 0, the binary array will be rotated to the right by count bits in the form of a linked list. If the remainder is 1, the binary array will be rotated to the left by count bits in the form of a linked list. The second step uses logical mapping to generate two encryption matrices of the same size as the length and width of the image, and then performs two XOR operations with the values of pixels on the image to obtain the encrypted image.

Four images are encrypted using both existing algorithms and the proposed scheme. The resulting data are presented in Table 1. From Table 1, it can be seen that the performance of the proposed algorithm can meet real-time requirements. Table 1 also displays the run times of other related algorithms for comparison.

From Table 1, it can be observed that the entropy value of the proposed selective encryption algorithm is lower than that of the algorithms in [24,25]. However, in terms of encryption speed, the proposed algorithm significantly outperforms the related algorithm. Specifically, it takes only about 0.8 s to encrypt an image using the proposed algorithm, whereas Rostami’s scheme requires approximately 46 s of computing time and Diaconu’s algorithm demands 177 s. Through a comparative analysis of these two parameters, it is evident that the proposed algorithm offers a significant improvement in running time compared to the related algorithms.

The JWE scheme presented in this work incorporates GoL permutation, bit-XOR, and SVD diffusion. The joint encryption/watermarking algorithm is expected to be faster than existing schemes due to parallel processing capabilities. In this technique, bit-XOR and SVD computations are reversible, allowing them to meet multi-level security requirements. The proposed JWE method addresses performance issues by applying permutation and SVD diffusion to selected key content. DWT enables all security actions to run in parallel, further enhancing efficiency. Diffusion based on bit-XOR and SVD computations increases the security level to meet various requirements. Since bit-XOR and SVD operations are reversible, decryption will be swift and efficient.

#### 4.6.2. Algorithm Comparison

The algorithm in [31] performed SVD on the horizontal and vertical components within the DWT domain, and then the watermark image was divided into two equal parts. They were multiplied by *K*, which is an increment factor. These two products were then added to the two singular value vectors. The watermarked content was obtained through the inverse DWT process. The normalized correlation (NC) values obtained are shown in Table 2. However, the method in [31] is less robust against noise and cropping attacks compared to the proposed watermarking scheme. Both security algorithms have their respective advantages and disadvantages, and different watermarking algorithms can be tailored for use in various fields.

Table 3 summarizes the proposed security methods alongside some existing approaches including the watermarking scheme in [44], the joint encryption and watermarking scheme in [45], and the multi-security level scheme [46]: the hybrid watermarking and encryption technology [47], the image encryption algorithm [15], the watermarking method [14], and the multi-level security algorithm [31]. In Table 3, selective encryption refers to the practice of encrypting only the most important parts of the data, rather than all coefficients.

These related security schemes are not scalable for protecting social images on multimedia social platforms. JPEG images are predominantly distributed on these platforms. However, these existing security methods do not offer comprehensive protection throughout the entire lifecycle of social images. Sharing images in the spatial domain can lead to multimedia big data issues on multimedia social platforms, causing significant computational and communication burdens due to the large number of uncompressed images involved.

The proposed JWE method is scalable for secure sharing of social JPEG images. By employing selective encryption and fast DWT transformation from DCT blocks, the method not only secures multimedia content but also reduces time complexity.

## 5. Conclusions

This paper proposes a social JPEG image security algorithm utilizing JWE. The primary objective is to ensure the information security of multimedia content on social multimedia platforms and facilitate safe sharing of social images through watermarking and encryption technology. This method offers an efficient content sharing approach with low time complexity. It guarantees the security of the entire distribution process, with watermarking technology used to authenticate copyright and fingerprint technology enabling active traceability.

With the proposed JWE method, the selective high-bit plane encryption algorithm for the LL, HL, and LH sub-bands includes scrambling with block GoL, diffusion with bit-XOR, and SVD computing. Selective permutation further meets fast and secure requirements. The embedding of a watermark addresses the copyright issue of images, while image encryption technology enhances the security of image information for secure communication on multimedia social platforms. The combined use of watermarking/fingerprinting and encryption ensures a dual level of security. Ultimately, our security scheme achieves secure multimedia content distribution in social multimedia platforms.

In the future, the algorithm needs further optimization to enhance its resistance to various attacks and improve encryption performance. This will strengthen the security of social image sharing on multimedia social platforms, mitigate the high burdens of computing and storage resources, and prevent information leakage caused by malicious attacks. We plan to study the JWE algorithm for JPEG 2000 and extend it to audio and video watermarking to improve the security of multimedia sharing in social networks.

## Figures and Tables

**Table 1:** Encryption performance and entropy analysis.

Image	Proposed		[24]		[25]		[32]	
	Entropy	Time (s)	Entropy	Time (s)	Entropy	Time (s)	Entropy	Time (s)
Plane	6.5628	0.7694	7.999724	44.3256	7.999715	168.352	5.3678	0.8935
Baboon	6.6258	0.7928	7.999745	45.3287	7.999716	176.239	5.2336	0.8637
Watch	6.1246	0.7593	7.999769	43.2687	7.999782	168.325	5.4263	0.8691
Peppers	6.3926	0.7789	7.999769	46.2189	7.999763	158.326	5.1256	0.8536

**Table 2:** Watermark NC value.

Attack	Plane		Baboon		Watch	
	Proposed	[31]	Proposed	[31]	Proposed	[31]
No attack	0.9996	0.9989	0.9962	0.9908	0.9962	0.9993
Upper left corner cropping (1/16)	0.9345	0.5523	0.8235	0.2912	0.9796	0.8965
Center cropping (1/16)	0.9652	0.3158	0.8291	0.1891	0.9548	−0.0217
Around cropping (1/8)	0.6613	−0.1561	0.8039	0.1726	0.9537	−0.3612
Salt and pepper (0.005)	0.8435	0.8028	0.8863	0.8327	0.8996	0.8938
Salt and pepper (0.01)	0.7929	0.7102	0.8192	0.6958	0.8765	0.7821
Salt and pepper (0.02)	0.7682	0.5123	0.8186	0.6128	0.8798	0.5587
Gaussian noise (0.001)	0.7956	0.6127	0.8278	0.5736	0.8537	0.4367
Gaussian noise (0.005)	0.7893	0.6539	0.8293	0.6312	0.8856	0.5129
Gaussian noise (0.01)	0.7639	0.6678	0.8369	0.6218	0.8678	0.5357

**Table 3:** Comparisons of the related schemes.

	Ours	[45]	[44]	[46]	[15]	[47]	[14]	[31]
Watermarking	Yes	Yes	Yes	Yes	No	Yes	Yes	Yes
Selective encryption	Yes	No	No	No	No	No	No	No
Tracing	Yes	Yes	No	Yes	No	Yes	Yes	Yes
Scalability	Yes	No	No	No	No	No	No	No
Encryption domain	DWT/DCT	Spatial	Spatial	Spatial	Spatial	No	No	Spatial
Encryption scheme	Chaos	RC4	RC4	Chaos	Chaos	No	No	Chaos
Watermark domain	DWT	Spatial	Spatial	DWT/DCT	No	FRFT	DWT/DCT	DWT
Watermark detection	Encrypted	Plaintext	Plaintext	Plaintext	No	Plaintext	Plaintext	Plaintext
Controllable security level	Yes	No	No	No	No	No	No	No

## Data Availability

The data generated and analyzed in this study are included in this published article.

## Abbreviations

The following abbreviations are used in this paper:

GoL	Game of Life
SVD	Singular value decomposition
DWT	Discrete wavelet transform
JWE	Joint watermarking and encryption
SHA	Secure hash algorithm
DPV	Discrete point vector
HNN	Hopfield neural network
QIM	Quantization index modulation
FRFT	Fractional Fourier transform
TSH	Tree structure Haar
SNA	Social network analysis
NC	Normalized correlation
